# Study on the inhibition of *Mfn1* by plant-derived *miR5338* mediating the treatment of BPH with rape bee pollen

**DOI:** 10.1186/s12906-018-2107-y

**Published:** 2018-01-30

**Authors:** Xuan Chen, Ren-zhao Wu, Yong-qiang Zhu, Ze-ming Ren, Ye-ling Tong, Feng Yang, Guan-hai Dai

**Affiliations:** Institute of Basic Medicine, Zhejiang Academy of Traditional Chinese Medicine, No. 132, Tianmushan Road, Xihu District, Hangzhou, Zhejiang China

**Keywords:** Benign prostatic hyperplasia, m*iR5338*, *Mfn1*, Rape bee pollen, Cross-kingdom

## Abstract

**Background:**

Recent studies have found that plant derived microRNA can cross-kingdom regulate the expression of genes in humans and other mammals, thereby resisting diseases. Can exogenous miRNAs cross the blood-prostate barrier and entry prostate then participate in prostate disease treatment?

**Methods:**

Using HiSeq sequencing and RT-qPCR technology, we detected plant miRNAs that enriched in the prostates of rats among the normal group, BPH model group and rape bee pollen group. To forecast the functions of these miRNAs, the psRobot software and TargetFinder software were used to predict their candidate target genes in rat genome. The qRT-PCR technology was used to validate the expression of candidate target genes.

**Results:**

Plant *miR5338* was enriched in the posterior lobes of prostate gland of rats fed with rape bee pollen, which was accompanied by the improvement of BPH. Among the predicted target genes of *miR5338*, *Mfn1* was significantly lower in posterior lobes of prostates of rats in the rape bee pollen group than control groups. Further experiments suggested that *Mfn1* was highly related to BPH.

**Conclusions:**

These results suggesting that plant-derived *miR5338* may involve in treatment of rat BPH through inhibiting *Mfn1* in prostate. These results will provide more evidence for plant miRNAs cross-kingdom regulation of animal gene, and will provide preliminary theoretical and experimental basis for development of rape bee pollen into innovative health care product or medicine for the treatment of BPH.

**Electronic supplementary material:**

The online version of this article (10.1186/s12906-018-2107-y) contains supplementary material, which is available to authorized users.

## Background

Recent studies have found that plant derived microRNA (miRNA) can cross-kingdom regulate the expression of genes in humans and other mammals, and affect the physiological and pathological processes of mammals [1]. The miRNAs are a kind of small RNA with a length of about 22 nucleotides. They participate in many key biological processes by regulating the expression of genes at post transcriptional levels [2]. Zhang et al. first found that exogenous plant miRNA named *miR168a* could inhibit translation of mammalian low-density lipoprotein receptor adapter protein 1 in liver, and consequently decrease low-density lipoprotein removal from mouse plasma [1]. After that, several groups of researchers have detected multiple plant miRNAs in the blood, milk, organs, and feces of human and other mammals [3–6]. It is noteworthy that certain plant miRNAs, after entering mammalian cells, can specifically resist influenza viruses [7], colon cancer [8], breast cancer [9] and other diseases.

Pollen is the male gametophyte of sexual reproduction of flowering plants. Pollen often collected by bees, so usually named bee pollen. Rape bee pollen is the highest yield bee pollen in China, and it is the raw materials of prostate drug named “Qian Lie Kang” which is used to improve prostate diseases. We previously reported that miRNAs in rape bee pollen could be absorbed into the blood of mice [10]. While the randomly chosen *miR169j* and *miR167b* were not detected in mouse prostate, the reason might be that certain organs absorb certain kind of exogenous miRNAs, and randomly chosen miRNA can not be taken in prostate. So, whether miRNAs involved in diet could cross the blood-prostate barrier [11] and entry prostate then participate in prostatic disease treatment? No related results have been reported so far.

In the present study, we detected whether plant miRNA could be absorbed by prostate and could participate in the treatment of benign prostatic hyperplasia (BPH) in rats.

## Methods

### Rat experiments

Adult male SD rats (6 weeks) weighing approximately 200 g were bought from Shanghai sirc laboratory animal Co. Ltd. They were housed under standard conditions. All of the animal experiments were conducted with the approval of the Animal Experimentation Ethics Committee of the Zhejiang Academy of TCM (No:[2016]040, Zhejiang, China).

Six rats were randomly divided into normal group, and the rest rats were received 2 mg/kg testosterone propionate (TP, Shanghai general pharmaceutical CO., LTD, China) by intraperitoneal injection for 14 days. After anatomical determination, the BPH rats were randomly divided into rape bee pollen group and model group (*n* = 6). The rape bee pollen was made into suspensions (0.639 g/mL). Then rats in rape bee pollen group were gavage fed (10 mL/kg) with the suspensions (final rape bee pollen: 6.39 g/kg) and rats in normal group were gavage fed with clean water (10 mL/kg). Meanwhile, the TP dosage was reduced to half (1 mg/kg) for another 3 weeks. Rats in normal group received olive oil accordingly (2 mg/kg for the first 2 weeks, then 1 mg/kg for another 3 weeks). Weight of each rat was recorded twice a week. Two hours after last injection, each rat was anesthetized by 50 mg pentobarbital sodium (2%). Then each lobe of prostate was dissected and weighed. Then we dissected and frozen all organs of each rat for further study, so all rats were sacrificed on test-bed.

### Deep sequencing and bioinformatics analysis

The sequencing procedure was conducted in total RNA extracted from posterior lobes of rats using the Trizol Reagent (Invitrogen, Carlsbad, CA, USA) according to the manufacturer’s instructions. Then small RNA (18-30 nt) were fractionated and recovered by the PAGE, and ligated with 3′ adaptor ligation and 5′ adaptor ligation, then reverse transcription to cDNA and cloned by PCR. The PCR products in the range 62~ 75 nt were recovered and purified by PAGE and used for library construction. Agilent 2100 Bioanaylzer and ABI StepOnePlus Real-Time PCR System were used in quantification and qualification of the sample library. At last, the library was sequenced using HiSeq sequencing system.

Tags from Hiseq sequencing went through the data cleaning analysis to get credible clean tags. Then the length distribution of the clean tags and common and specific sequences between samples were summarized. Then the standard analysis annotated the clean tags into different categories and taken those which can not be annotated to any category to predict the novel miRNA using Mireap or Mirdeep. Then targets of each miRNA were predicted using psRobot software and TargetFinder software.

### RT-qPCR

Total RNA of each sample was extracted using Trizol® Reagent (ambion® manufactured by Life technologiesTM, USA) according to the instruction with slightly modifications. 1 mL of 100% isopropanol was added instead of 0.5 mL to make precipitation of miRNAs more complete. Accordingly, the pellet was washed with 75% ethanol twice instead of once to purify the sediment.

The reverse transcription reactions of mRNA (500 ng total RNA) was performed using PrimeScript™ RT Master Mix (Takara, Japan) and miRNA (500 ng total RNA) using Mir-X miRNA First Strand Strand Synthesis Kit (Takara, Japan) respectively. The qPCR reactions were performed in StepOnePlus (Theremofisher, USA) using SYBR Premix Ex Taq II (Tli Rnase H Plus) (Takara, Japan). Primers were designed and synthesized by Shanghai Sangon Biotech (China). The forward primer for *miR5338*: ATCTTTGCCGGGTGTCTCTGAC; for *miR894*: CGTTTCACGTCGGGTTCACC; the reference gene for miRNA was u6 (contained in the miRNA RT kit). The primers for *Mfn1*: Forward: CACAGAGCTGGACATCTGGA, reverse: AGCCGCTCATTCACCTTATG; *Armc10*: Forward: AATCCAGCCATGACAGAAGG, reverse: CCTTCCACTCTGAGGCAGTT; *Rhobtb3*: Forward: GACATACCAAGCCAGGAAGC, reverse: TGGCACAGTTGCTCCTTGTA; *Nme4*: Forward: GGACCTACAGAGGAAGCCATT, reverse: AGTCCGTGTGTCCTATCATGG; *Efhc1*: Forward: TGTCTGTCATCGAGCCTGTC, reverse: AGGTTGATTCCACGGTTCAG; *Lrit2*: Forward: AGTGACCAACCTTGCTGGAG, reverse: GAGGAGGTCATCCACAGCAT; *Pfkfb1*: Forward: TAGCCAACTTCATCCGGTCT, reverse: GGCCTTCCACTGTTCATAGG; *Htr2c*: Forward: GGCATACCAATGAACGTGTG, reverse: AATCCTCTCGCTGACCACAT; *Pemt*: Forward: TGGAATGTGGTAGCAAGGTG, reverse: GAGCGGAGGATGTTCAGAAG; *Slc7a7*: Forward: CTTCCATTGTGGCTGCTTCT, reverse: AGAAGGCACTGGTGTGAACC; The reference gene for mRNAs was *EF-1a*: Forward: CGAGCCACCATACAGTCAGA, reverse: CCATTCCAACCAGAAATTGG.

### Statistical analyses

SPSS statistics 17.0 was used to analyze the data. The body weight, lobe index and gene expression level among three groups were analyzed using One Way Analysis of Variance, and between two groups were analyzed using Student’s test. Differences are considered statistically significant at *P* < 0.05.

## Results

### Effects of rape bee pollen on body weight and prostate index of each lobe in BPH rats

#### Effects of rape bee pollen on body weight in BPH rats

To evaluate whether rape bee pollen could affect the body weight of rats, we weighted the rat body weight twice a week. By variance analysis, rape bee pollen had no significant effect on body weight of rats (Table 1).

**Table 1:** The effects of rape bee pollen on body weight in rats

Groups	d1 (mean ± SD)	d 30 (mean ± SD)
Normal group	234.40 ± 11.91	328.47 ± 29.12
Model group	234.46 ± 7.87	326.53 ± 28.71
Rape bee pollen group	238.71 ± 7.18	329.47 ± 27.46
Variance analysis	F = 0.000,*P* = 1.000	F = 0.183, *P* = 0.834

#### Effects of rape bee pollen on prostate index of each lobe in BPH rats

**Fig. 1:**
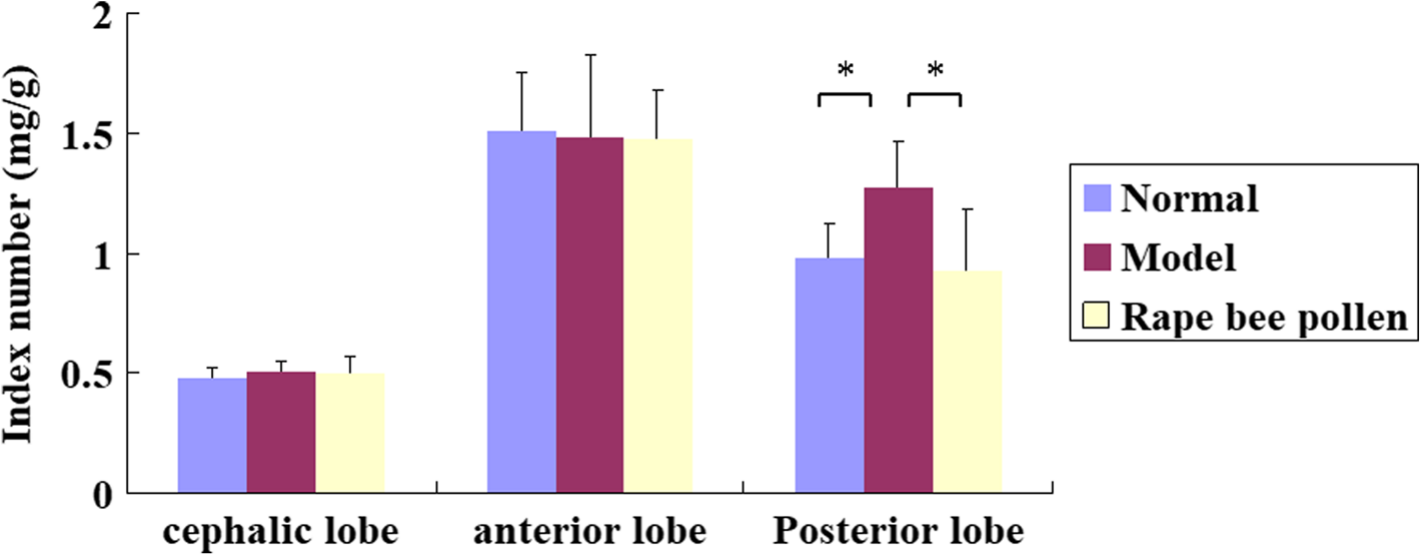
Effects of rape bee pollen on prostate index of each lobe in BPH rats induced by TP

Variance analysis was used to determine whether there were differences among the normal group, the model group and the rape bee pollen group in the index of cephalic lobes, anterior lobes and posterior lobes. The results showed that there were neither difference in cephalic lobes (F = 1.016,*P* = 0.384) nor in anterior lobes (F = 0.019, *P* = 0.981) among three groups (Fig. 1.). While, posterior lobes were significantly increased by BPH modeling, and decreased to normal level when treating with rape bee pollen (Fig. 1.) (F = 4.912, *P* = 0.022; normal vs model: *P* = 0.024;model vs rape bee pollen:*P* = 0.010; normal vs rape bee pollen: *P* = 0.748).

### The plant miRNAs enriched in posterior lobes of rats after intragastric administration of rape bee pollen

In order to detect whether the plant miRNAs are enriched in the posterior lobe of the prostate in rats administrated with rape bee pollen, we extracted total RNA of each posterior lobe of each rat from the model group and the rape bee pollen group, then combined into one sample separately for RNA-Seq. The results showed that abundance of 7 plant miRNAs were increased in the rape bee pollen group compared with the model group, including *miR894, miR5338, miR3440-5p, miR2878-5p, miR7754-5p, miR5015,* and *miR7731-3p*.

**Fig. 2:**
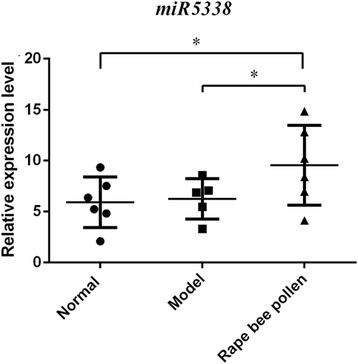
The abundance difference of miR5338 in posterior lobes in rats among rape bee pollen group and control groups

Using the qRT-PCR technology, we compared the abundance difference of the top 2 enriched miRNAs, *miR894* and *miR5338* among the normal group, model group and rape bee pollen group. After variance analysis, the results showed that the abundance of *miR5338* in the posterior lobe of the prostate in the rape bee pollen group was significantly higher than in the groups did not feed with rape bee pollen, that are the model group and the normal group (F = 5.396, *P* = 0.02;rape bee pollen group VS model group:*P* = 0.019; rape bee pollen group VS normal group: *P* = 0.010). No surprisingly, the abundance of *miR5338* was no difference between normal group and model group (*P* = 0.826). These results suggested that after administrated with rape bee pollen, plant *miR5338* can be enriched in the prostate gland of rats (Fig. 2). However, *miR894* has no difference among three groups.

**Fig. 3:**
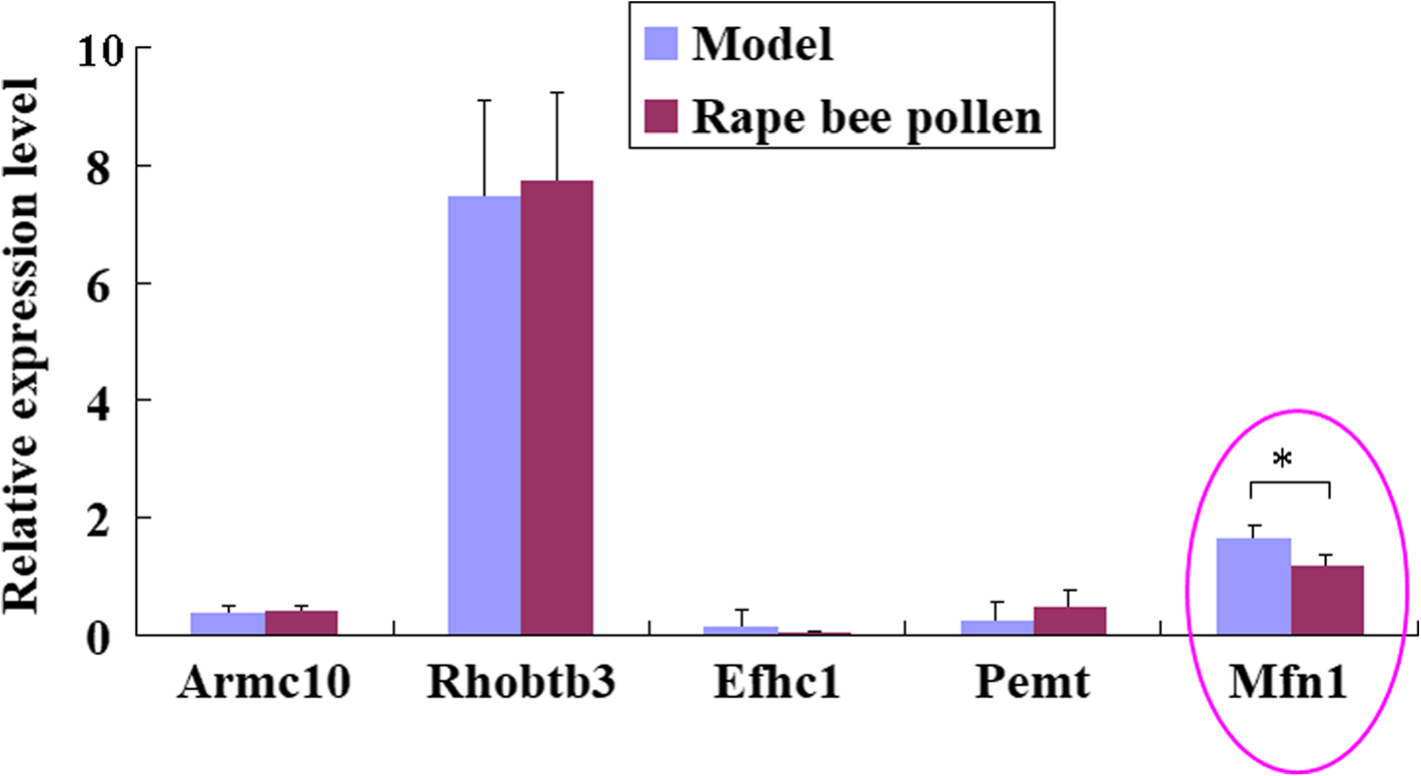
Comparison of abundance of candidate target genes of miR5338 in posterior lobes of prostates of rats between rape bee pollen group and model group

To investigate the role of *miR5338* in inhibiting BPH, we used psRobot and TargerFinder software to predict its target genes in the rat genome. As a result, we obtained 17 candidate target genes, including *Armc10, Rhobtb3, Pappa1, Nme4, Efhc1,Lrp1, Opn3, Lrit2, Pfkfb1, Htr2c, Pemt, Slc7a7, Mfn1, mirlet7f1, mir3596a, mir3596b,*and *mir3596c*. According to gene functions, we screened *Armc10, Rhobtb3, Nme4, Efhc1, Lrit2, Pfkfb1, Htr2c, Pemt, Slc7a7* and *Mfn1* to be validated further. Using the RT-qPCR technology, we tested the primer specificity of these genes. As a result, *Nme4, Efhc1, Lrit2, Pfkfb1, Htr2c,* and *Slc7a7* were eliminated due to a bad primer melt curve (Additional file 1), and *Armc10, Rhobtb3, Pemt* and *Mfn1* showed a qualified melt curve (Additional file 1) and a trend of differential expression between a mixed sample of the model group and the rape bee pollen group (Additional file 2). The difference in expression level of *Armc10, Rhobtb3, Pemt* and *Mfn1* in posterior lobes was further tested in rats of these two groups individually. By *t test*, only *Mfn1* was significantly lower in posterior lobes of rats in the rape bee pollen group than the model group (*P* = 0.006) (Fig. 3), suggesting that *miR5338* may involve in treatment of BPH through inhibiting *Mfn1*.

**Fig. 4:**
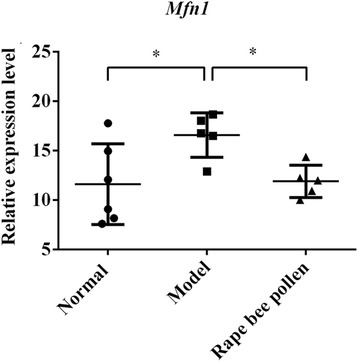
Comparison of abundance of Mfn1 between rape bee pollen group and control groups

While, is there a connection between *Mfn1* and BPH? We detected the difference in expression of *Mfn1* among the normal group, model group and rape bee pollen group by RT-qPCR technique and variance analysis, the results showed that the difference was significant (F = 4.568,*P* = 0.031) (Fig. 4). *Mfn1* was significantly increased in the model group than in the normal group (*P* = 0.016), then significantly reduced in rape bee pollen group than model group (*P* = 0.027), and tending to normal level (*P* = 0.871). This indicating that the elevated expression of *Mfn1* may be associated with BPH, and the enrichment of *miR5338* through oral taken rape bee pollen may involve in improvement of BPH through inhibiting *Mfn1* in prostate.

**Fig. 5:**
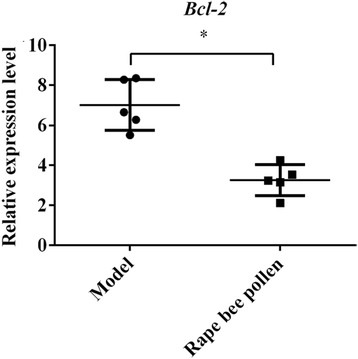
Comparison abundance of Bcl-2 between rape bee pollen group and model groups

To detect whether the accumulation of *miR5338* and down-regulation of *Mfn1* in posterior lobes of rats, both simultaneously induced by rape bee pollen, might be related to apoptosis of BPH cells, we compared the expression of *Bcl-2* between the model group and the rape bee pollen group by RT-qPCR (Fig. 5). By *t test*, *Bcl-2* was significantly reduced in the rape bee pollen group than in the model group (*P* = 0.000), hinting that *Bcl-2* might be a link in the signaling pathway of treatment of BPH through cell apoptosis, which might be induced by inhibition of *Mfn1* by *miR5338*.

## Discussion

Screening by deep-sequencing, we found that plant-derived *miR5338* was enriched in posterior lobes of rat prostates after administration of rape bee pollen. Is *miR5338* an executive of rape bee pollen in the treatment of prostate diseases? Through bioinformatics prediction and experimental verification, the candidate target gene of *miR5338*, *Mfn1* has attracted our attention.

Mitochondria are organelles with double membrane structure. Besides providing energy for cells, mitochondria are involved in the processes of cell differentiation, proliferation, apoptosis, cycle, and information transmission [12–14]. The realization of these functions requires the constant fusion and division of membrane structures between different mitochondria, which are precisely regulated by fusion and division, and imbalance leads to disease [15–17]. Mitochondrial fusion requires fusion proteins (*Mfn*) as vectors, and outer membrane fusion requires Mfn1 and Mfn2, and intimal fusion requires OPA1 [18, 19]. Abnormal expression of *Mfn* is involved in many diseases, including a variety of cancers, like prostate cancer [13, 16].

BPH is a common and frequently occurring disease in middle-aged and elderly men. The main pathogenesis is the loss of balance between proliferation and apoptosis of prostate cells under the stimulation of androgen and aging. Mitochondria bear the main switch role in cell apoptosis, and the fusion related gene *Mfn1* is closely related to apoptosis, and by interacting with genes such as *Bax, Bak, cytochrome C* and other genes in the apoptotic signaling pathway, *Mfn1* over-expression inhibits apoptosis and low expression promotes apoptosis [20].

Whether *Mfn1* is related to BPH has not been reported. But researchers have found that mitochondria in the prostate cells of BPH rats differ in shape from those in the normal group [21]. Is this related to the abnormal expression of the *Mfn1* gene that directly affects mitochondrial morphology? Our results showed that the expression of *Mfn1* in the posterior prostate gland of BPH rats was significantly higher than that in the control group, and decreased to normal level after treatment with rape bee pollen. We speculated that over-expression of *Mfn1* leads to morphological changes of mitochondria and participated in the development of BPH, and after administrated with rape bee pollen, *miR5338* was enriched in the prostate gland, inhibiting the expression of *Mfn1* and treating BPH. If the speculation was correct, what was the molecular basis for *Mfn1* on treatment of BPH? Is it related to apoptosis? Through the experiments, we found that rape bee pollen can significantly reduce the expression level of *Bcl-2* in the prostate gland of BPH rats. *Bcl-2* is an integral protein in the outer mitochondrial membrane, and also a gene in the apoptotic signaling pathway, which is highly expressed in BPH [22]. Over-expression of *Bcl-2* could increase mitochondrial size and inhibit cell apoptosis [23].

Based on this, we speculated that the increased expression of *Mfn1* in prostate promoted mitochondrial fusion, altered mitochondrial morphology, inhibited apoptosis in the occurrence and development of BPH; After administrated with rape bee pollen, plant source *miR5338* was enriched in the prostate, combined to *Mfn1* mRNA, reduced the expression level of *Mfn1*, inhibited mitochondrial fusion, reversed mitochondrial morphology, promoted cell apoptosis, improved BPH.

In the future, experiments should be performed to detect whether *Mfn1* is directly targeted by *miR5338* through dual luciferase reporter gene assay; to investigate whether enrichment of plant-derived miRNAs and decrease of *Mfn1* in prostate, both induced by rape bee pollen, were associated with changes in mitochondrial shape and gene expression level in apoptosis signaling pathway.

## Conclusions

These results suggesting that plant-derived *miR5338* may involve in treatment of rat BPH through inhibiting *Mfn1* in prostate. These results will provide more evidence for plant miRNAs cross-kingdom regulation of animal gene, provide preliminary theoretical and experimental basis for development of rape bee pollen into innovative health care product or medicine for the treatment of BPH.

## Additional files

Additional file 1: Melt curves for each gene. (DOC 933 kb)

Additional file 2: Comparison of abundance of *Armc10, Rhobtb3, Pemt* and *Mfn1* in posterior lobes of prostates of rats between a mixed sample of rape bee pollen group and a mixed sample of model group. (JPEG 30 kb)

## Abbreviations

BPH: Benign prostatic hyperplasia
*Mfn1*: Mitochondrial fusion protein 1
miRNA: microRNA
TP: Testosterone propionate
