# Supplementary figures and images for: Study on the inhibition of Mfn1 by plant-derived miR5338 mediating the treatment of BPH with rape bee pollen
Source: BMC Complement Altern Med. 2018 Jan 30;18:38. doi: 10.1186/s12906-018-2107-y (PMC5791735; doi:10.1186/s12906-018-2107-y)

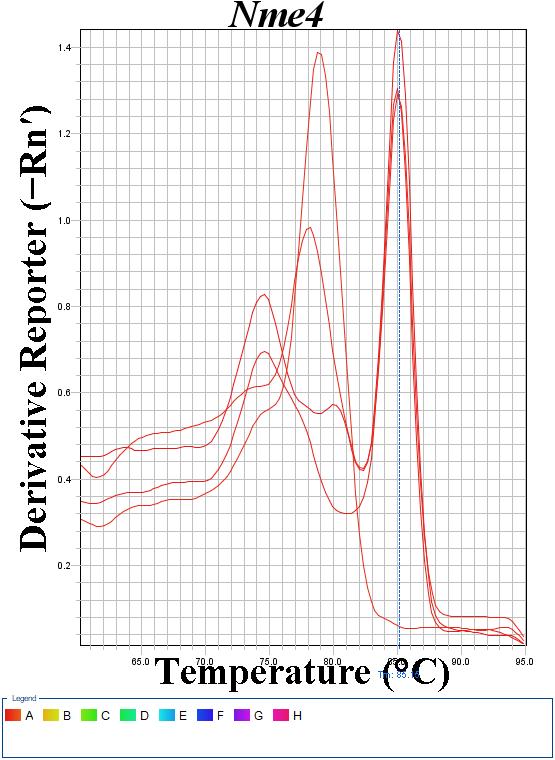

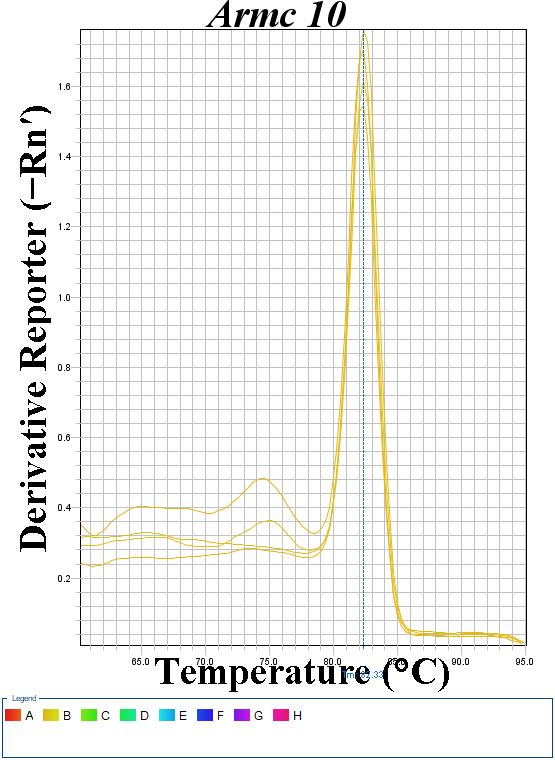

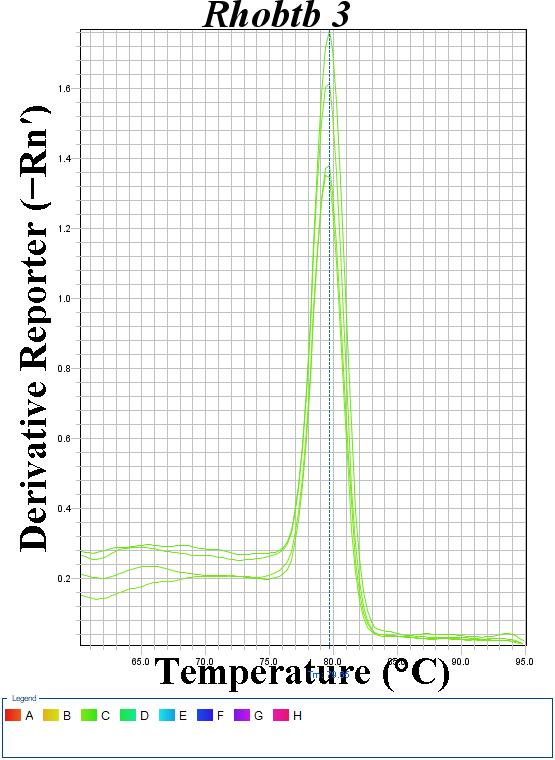


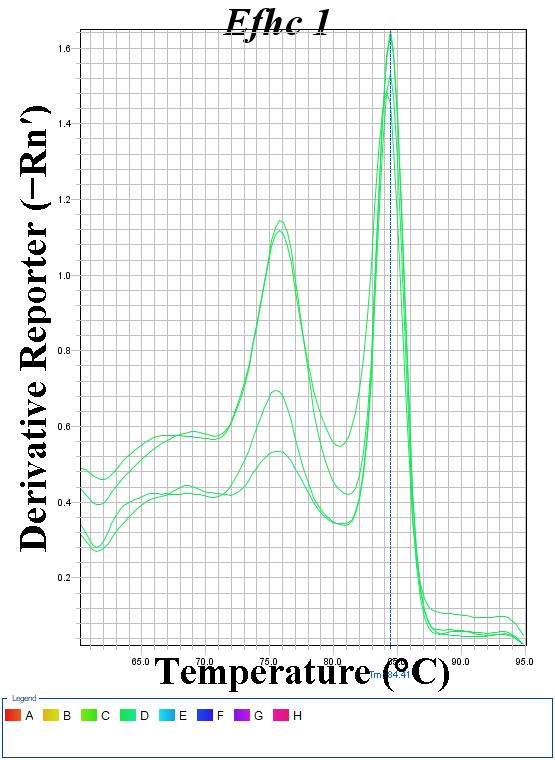

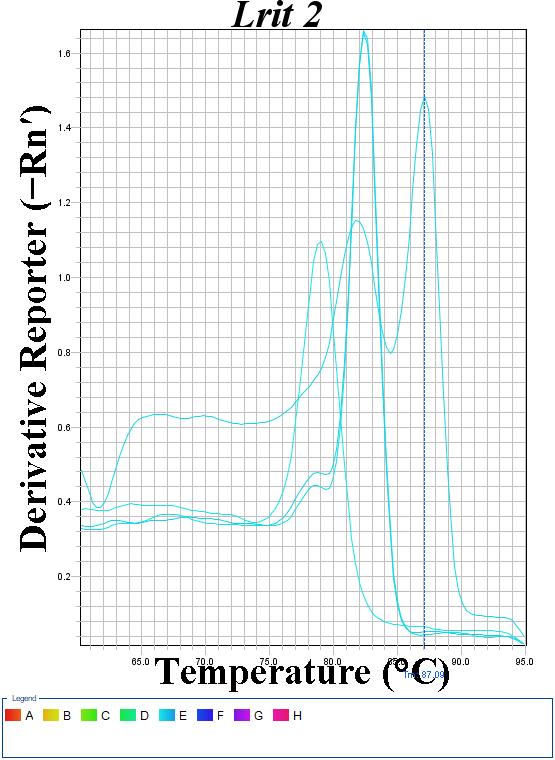

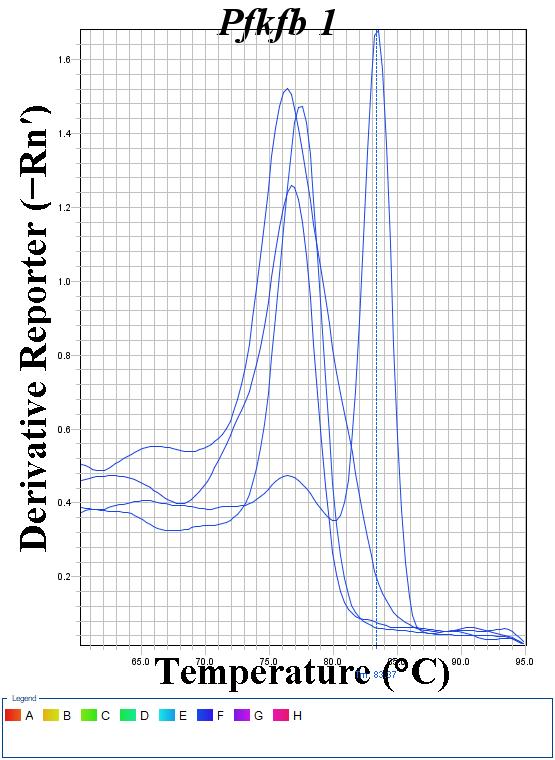


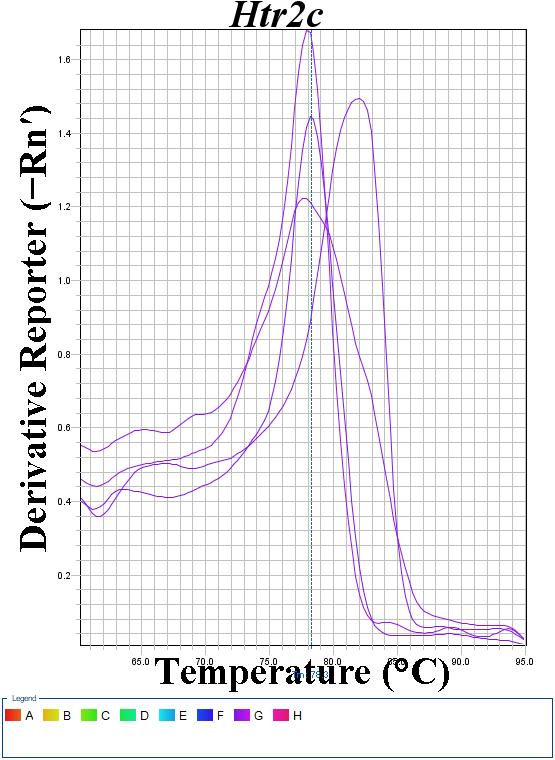

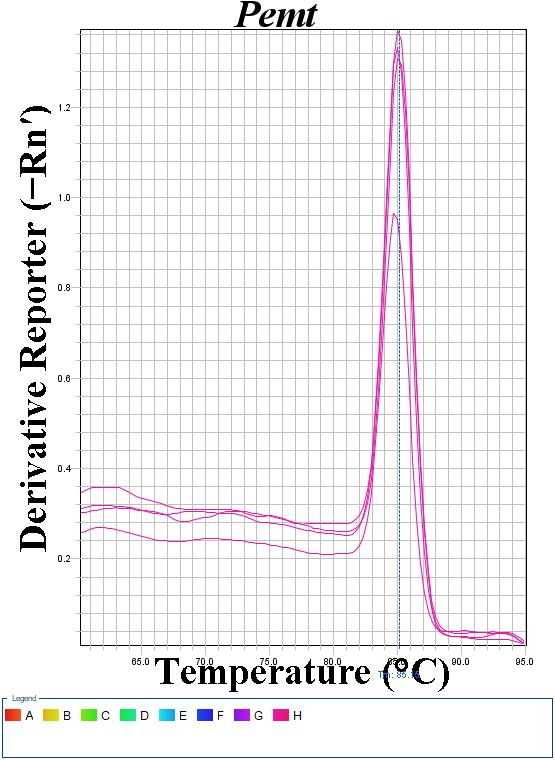

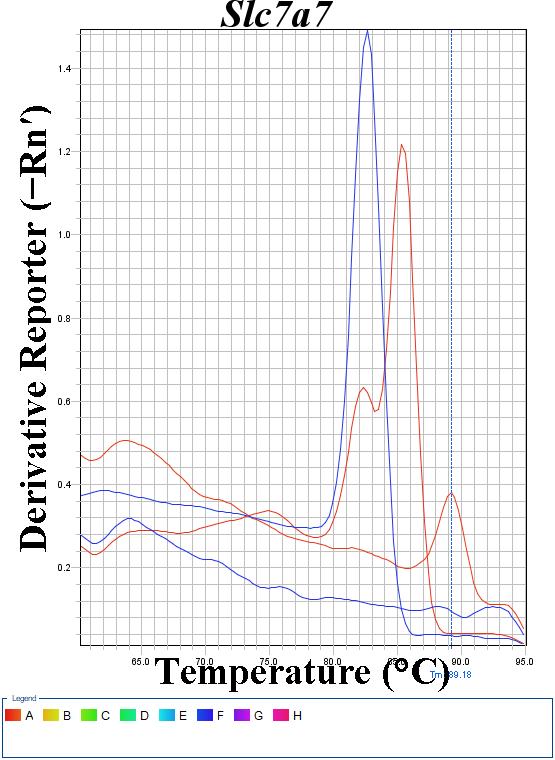


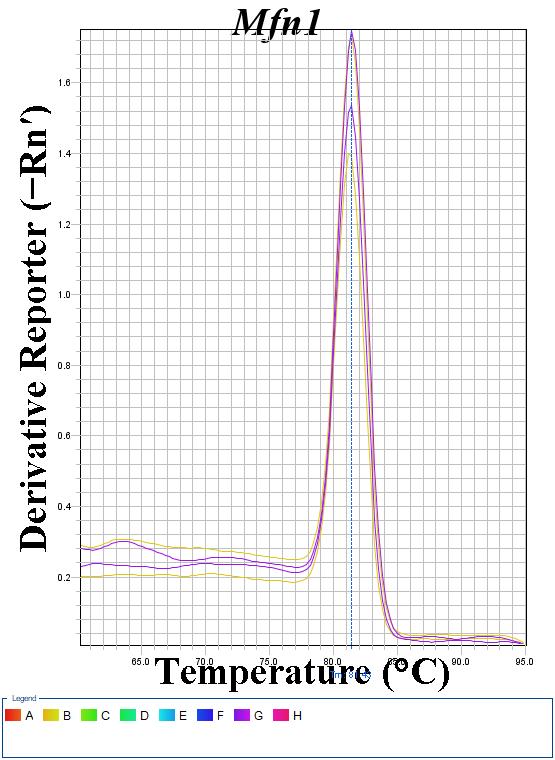

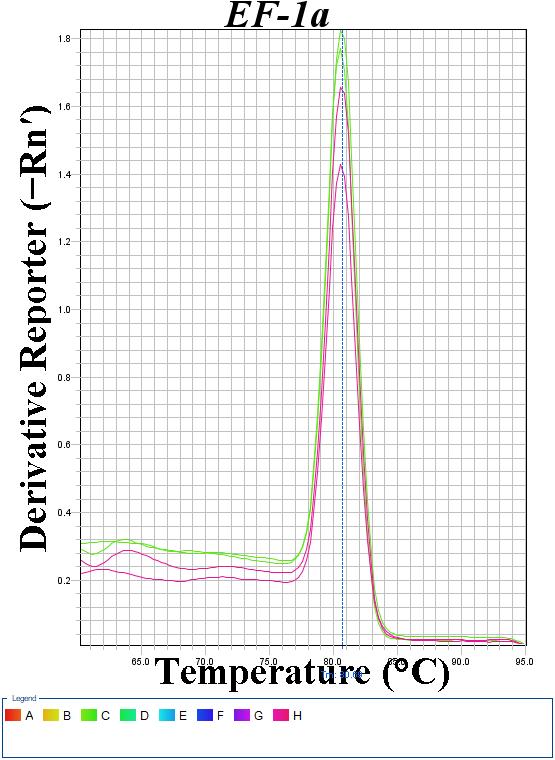


Figure S1 Melt curves for each gene

Supplement: Supplementary file 1 — Melt curves for each gene. (DOC 933 kb) [file 12906_2018_2107_MOESM1_ESM.doc]

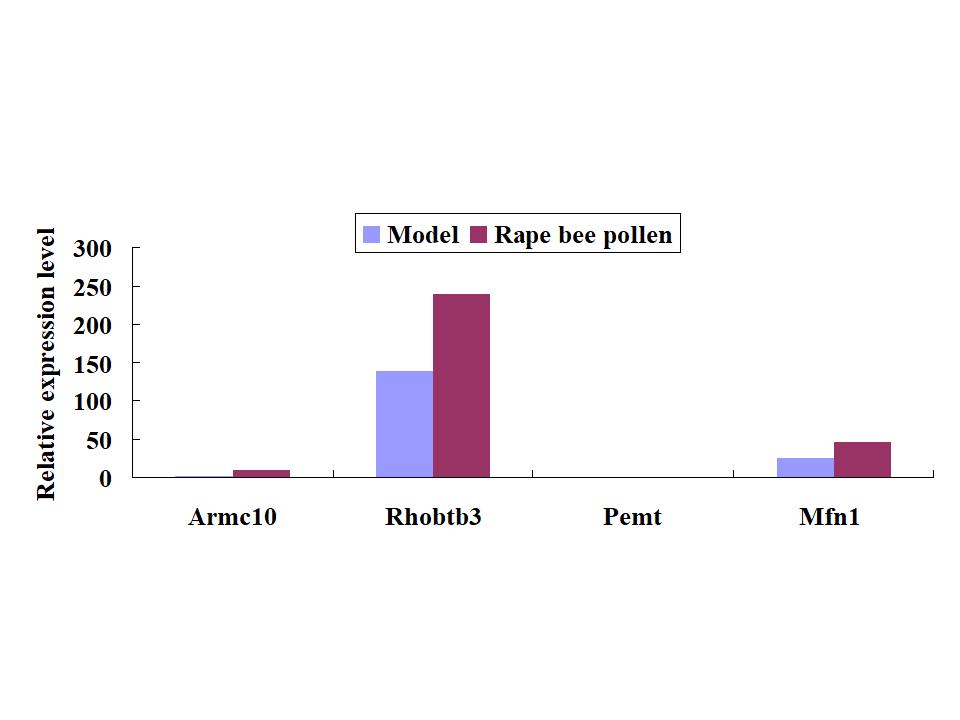

Supplement: Supplementary file 2 — Comparison of abundance of Armc10, Rhobtb3, Pemt and Mfn1 in posterior lobes of prostates of rats between a mixed sample of rape bee pollen group and a mixed sample of model group. (JPEG 30 kb) [file 12906_2018_2107_MOESM2_ESM.jpg]
